# Ocular Monkeypox — United States, July–September 2022

**DOI:** 10.15585/mmwr.mm7142e1

**Published:** 2022-10-21

**Authors:** Shama Cash-Goldwasser, Sarah M. Labuda, David W. McCormick, Agam K. Rao, Andrea M. McCollum, Brett W. Petersen, James Chodosh, Catherine M. Brown, Suk Yin Chan-Colenbrander, Caitlin M. Dugdale, Michael Fischer, Amy Forrester, Jayne Griffith, Rachel Harold, Bruce W. Furness, Vivian Huang, Aaron R. Kaufman, Ellen Kitchell, Rachel Lee, Nicholas Lehnertz, Ruth Lynfield, Ketzela Jacobowitz Marsh, Lawrence C. Madoff, Nelson Nicolasora, Dharmendra Patel, Roberto Pineda, Trey Powrzanas, Afsoon Roberts, Maria Teresa Seville, Ami Shah, Joshua M. Wong, Jana M. Ritter, Caroline A. Schrodt, Elliot Raizes, Sapna Bamrah Morris, Jeremy A. W. Gold, CDC Monkeypox Clinical Escalations Team, CDC Monkeypox Clinical Escalations Team, Amimah Asif, Amy Beeson, Ramon Bhatia, Brian F. Borah, Kevin Chatham-Stevens, Rewa Choudhary, Eleanor Click, Thomas D. Filardo, Romeo R. Galang, Julia Haston, Sophia Hsu, Gurpreet Kaur, Anne Kimball, James T. Lee, Grace Marx, Janet McNicholl, Maureen J. Miller, Rebecca Noe, Siobhan O’Connor, Kevin O’Laughlin, Kia Padgett, Gail Thompson, Farrell Tobolowsky, Isaac Zulu

**Affiliations:** ^1^CDC Monkeypox Emergency Response Team; ^2^Epidemic Intelligence Service, CDC; ^3^Minnesota Department of Health; ^4^University of New Mexico School of Medicine, Albuquerque, New Mexico; ^5^Massachusetts Department of Public Health; ^6^University of Minnesota Medical Center, Minneapolis, Minnesota; ^7^Massachusetts General Hospital, Boston, Massachusetts; ^8^Texas Department of State Health Services; ^9^Dallas County Health and Human Services, Dallas, Texas; ^10^District of Columbia Department of Health, Washington, D.C.; ^11^Maricopa County Department of Health, Phoenix, Arizona; ^12^Massachusetts Eye and Ear Infirmary, Boston, Massachusetts; ^13^University of Texas Southwestern Medical Center, Dallas, Texas; ^14^The George Washington University School of Medicine and Health Sciences, Washington, D.C.; ^15^Banner University Medical Center, University of Arizona, Phoenix, Arizona; ^16^Mayo Clinic Hospital, Phoenix, Arizona; ^17^Pueblo Family Physicians, Phoenix, Arizona.; CDC; CDC; CDC; CDC; CDC; CDC; CDC; CDC; CDC; CDC; CDC; CDC; CDC; CDC; CDC; CDC; CDC; CDC; CDC; CDC; CDC; CDC; CDC; CDC

As of October 11, 2022, a total of 26,577 monkeypox cases had been reported in the United States.* Although most cases of monkeypox are self-limited, lesions that involve anatomically vulnerable sites can cause complications. Ocular monkeypox can occur when *Monkeypox virus* (MPXV) is introduced into the eye (e.g., from autoinoculation), potentially causing conjunctivitis, blepharitis, keratitis, and loss of vision (*1*). This report describes five patients who acquired ocular monkeypox during July–September 2022. All patients received treatment with tecovirimat (Tpoxx)^†^; four also received topical trifluridine (Viroptic).^§^ Two patients had HIV-associated immunocompromise and experienced delays between clinical presentation with monkeypox and initiation of monkeypox-directed treatment. Four patients were hospitalized, and one experienced marked vision impairment. To decrease the risk for autoinoculation, persons with monkeypox should be advised to practice hand hygiene and to avoid touching their eyes, which includes refraining from using contact lenses (*2*). Health care providers and public health practitioners should be aware that ocular monkeypox, although rare, is a sight-threatening condition. Patients with signs and symptoms compatible with ocular monkeypox should be considered for urgent ophthalmologic evaluation and initiation of monkeypox-directed treatment. Public health officials should be promptly notified of cases of ocular monkeypox. Increased clinician awareness of ocular monkeypox and of approaches to prevention, diagnosis, and treatment might reduce associated morbidity.

During the 2022 multinational outbreak, CDC has provided consultation to clinicians treating patients with monkeypox.^¶^ This report describes demographic characteristics, clinical features, and outcomes as of October 11 for five patients who received a diagnosis of ocular monkeypox during July–September 2022. Ocular monkeypox was defined as the presence of new ocular disease compatible with *Orthopoxvirus* (OPXV) infection in a patient with probable or confirmed monkeypox** and no alternative explanation for the ocular disease. CDC obtained data during clinical consultation and worked with treating clinicians and jurisdictional health departments to follow patient progress. Patient permission for the use of the clinical image was obtained. This activity was reviewed by CDC and was conducted consistent with applicable federal law and CDC policy.^††^

## Patient A

**FIGURE 1:**
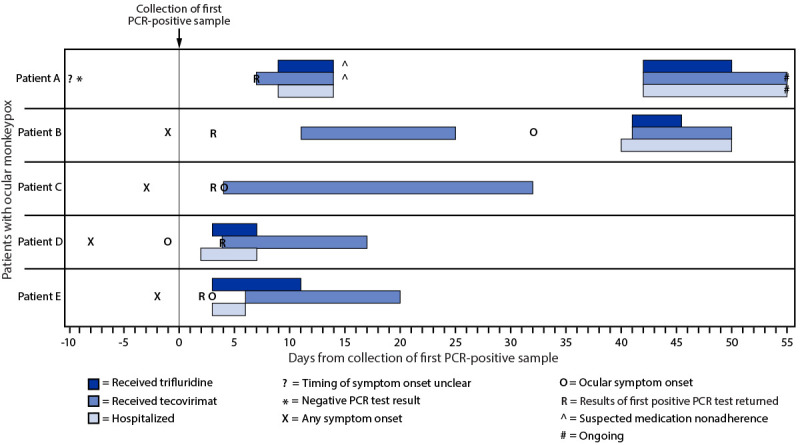
Timeline of testing, symptom onset, and initiation of medical countermeasures for patients with ocular monkeypox — United States, July–September 2022 **Abbreviation:** PCR = polymerase chain reaction.

**FIGURE 2:**
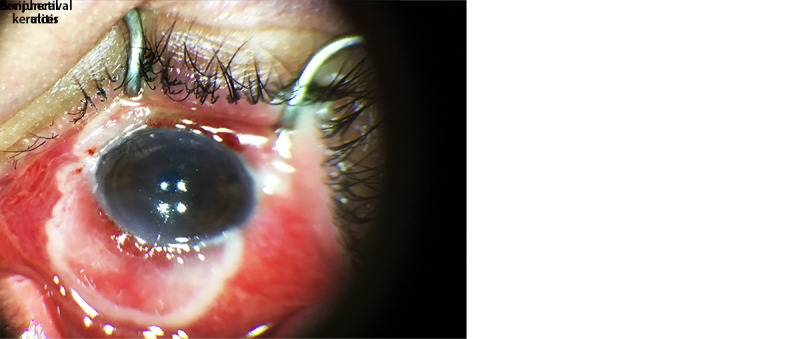
Left eye in a patient* with HIV-associated immunocompromise and ocular monkeypox, with conjunctivitis and conjunctival lesion earlier in the course of monkeypox illness (A), and with conjunctival ulcer and peripheral keratitis later in the course of monkeypox illness (B) — United States, August–September 2022 Photo A/Nathanael Adjei-Kyeremeh Photo B/Dharmendra R. Patel * Patient has consented to the publication of these photographs.

In August 2022, a man aged 20–29 years with HIV disease (Table) (Figure 1) (CD4 = 25 cells/mm^3^, not receiving antiretroviral therapy [ART]) was evaluated in an outpatient clinic for a rash on his buttocks, chest, arms, and hands that was compatible with monkeypox.^§§^ Swabs collected from lesions on his chest were sent for polymerase chain reaction (PCR) testing for OPXV, and results were negative. Ten days later, the patient presented for care again, this time with progressive rash as well as left eye symptoms, including pain, itching, swelling, discharge, foreign body sensation, photosensitivity, and vision changes. The rash was swabbed again to test for OPXV, and he was provided a referral to ophthalmology. Seven days later, PCR testing returned positive results for OPXV, and he commenced treatment with oral tecovirimat. Two days later, he was admitted to a hospital because of worsening ocular symptoms. Ophthalmologic exam was notable for left eye conjunctivitis. Visual acuity in the left eye was 20/40. The patient started intravenous tecovirimat and topical trifluridine drops to his left eye on admission, and he was initiated on ART. His ocular symptoms improved, and he was discharged after 5 days with prescriptions for oral tecovirimat, topical trifluridine, and ART. During the next 4 weeks he developed new facial lesions and decreasing left eye vision, for which he was readmitted to the hospital; health care providers suspected nonadherence with prescribed medications. Ophthalmologic examination revealed left eye conjunctivitis, keratitis, and a conjunctival ulcer (Figure 2). Left eye visual acuity was measured at 20/300. A swab of the conjunctival lesion yielded a positive PCR test result for OPXV, and biopsy showed necroulcerative conjunctivitis with extensive intralesional orthopoxviral antigen detected by immunohistochemistry. The patient was restarted on intravenous tecovirimat, and his left eye was treated with topical trifluridine for 1 week as well as topical povidone-iodine. Currently, the patient is on day 14 of intravenous tecovirimat and remains hospitalized for treatment of ocular monkeypox. He has experienced waxing and waning of left eye pain, irritation, and photosensitivity. Left eye visual acuity was most recently measured at 20/800 (profound visual impairment), although bedside visual acuity assessments have been challenging. His prognosis for vision recovery is currently unknown.

**TABLE:** Demographic characteristics, clinical characteristics, and outcomes among patients with ocular monkeypox — United States, July–September 2022

Patient	Age group, yrs, sex	HIV status (CD4 count, cells/mm^3^)	Signs and symptoms	Ocular exam findings	Outcomes
**A**	20–29, male	Positive (25)	Nonocular: rash on arms, hands, chest, and buttocks	Unilateral conjunctivitis, conjunctival lesion, and keratitis	Hospitalization: 22 days and ongoing
			Ocular: redness, pain, itching, swelling, discharge, foreign body sensation, photosensitivity, and vision changes		Vision impairment ongoing and treatment for active ocular monkeypox ongoing
**B**	30–39, male	Positive (78)	Nonocular: rash on face, chest, legs, and perianal area	Unilateral medial canthus lesion, conjunctivitis, conjunctival lesion, and corneal lesion	Hospitalization: 10 days
			Ocular: redness, pain, itching, and photosensitivity		No vision changes
**C**	30–39, male	Negative	Nonocular: rectal pain and perianal rash	Bilateral conjunctivitis	Hospitalization: none
			Ocular: redness, pain, and discharge		No vision changes
**D**	30–39, male	Negative	Nonocular: rash on penis, abdomen, and wrist	Unilateral eyelid lesion, conjunctivitis, conjunctival lesion, and preseptal cellulitis	Hospitalization: 5 days
			Ocular: redness, pain, and periorbital swelling		No vision changes
**E**	30–39, female	Negative	Nonocular: rash on vaginal labia, buttocks, back, chin, and forehead	Unilateral eyelid lesion, conjunctivitis, conjunctival lesion, and subconjunctival nodule	Hospitalization: 3 days
			Ocular: redness and pain		No vision changes

## Patient B

In July 2022, a man aged 30–39 years with HIV disease (CD4 = 78 cells/mm^3^, not on ART) was evaluated at an emergency department with a rash on his chest, legs, perianal area, and face, including on the bridge of his nose near his right eye (Table) (Figure 1). Swabs of lesions from his face and scalp were taken to test for OPXV, but because tecovirimat was not available in the emergency department, he was referred to an outpatient clinic to receive tecovirimat. The swabs tested PCR-positive for OPXV. The patient was evaluated at an outpatient clinic 9 days after testing and was prescribed ART and 14 days of oral tecovirimat. His rash began to resolve during treatment. Two weeks after completion of tecovirimat, he developed new and worsening facial lesions. The lesion on his nose expanded onto the right medial canthus and over the conjunctiva, and he experienced right eye redness, pain, itching, and photosensitivity, for which he was hospitalized. He did not experience vision changes. Ophthalmologic exam results were notable for right eye conjunctivitis, several small conjunctival nodular lesions, and corneal ulcers. He was treated again with intravenous tecovirimat for 10 days and with topical trifluridine drops for 5 days and antibacterial eye drops to the right eye. He was discharged upon regression of the eye lesion and improvement in conjunctivitis 10 days after admission, without further treatment for monkeypox.

## Patient C

In August 2022, a previously healthy man aged 30–39 years developed rectal pain and perianal lesions. He went to an emergency department 3 days later and swabs of those lesions were taken for OPXV testing (Table) (Figure 1). Three days later, when the swabs yielded positive PCR test results for OPXV, the patient was prescribed oral tecovirimat for rectal pain. Two days later, he was evaluated again in the emergency department with right eye pain, redness and discharge. He did not experience vision changes. Ophthalmologic exam was notable for right eye conjunctivitis. He subsequently developed bilateral conjunctivitis; the treating physicians suspected the patient had autoinoculated both eyes with MPXV by rubbing them. The patient’s bilateral conjunctivitis persisted for 3 weeks after resolution of his perianal lesions. The course of tecovirimat was extended until all ocular symptoms resolved, which occurred after 1 month of treatment.

## Patient D

In August 2022, a previously healthy man aged 30–39 years developed a groin rash (Table) (Figure 1). One week later, he was examined at an emergency department for right eye redness, pain, and eyelid swelling. He reported rubbing his right eye. Lesions were noted on his penis, abdomen, and one wrist. Samples were collected from the body lesions for OPXV testing; the patient received empiric treatment for gonorrhea and chlamydia. Providers attributed the eye symptoms to bacterial preseptal cellulitis and he was discharged on oral antibiotics. Two days later, the patient returned with multiple right eyelid lesions, periorbital swelling, and eye pain, for which he was admitted to a hospital. He did not experience vision changes. Ophthalmologic exam was notable for right eye conjunctivitis as well as four ulcers on the eyelid margin and three lesions on the palpebral conjunctiva, which were swabbed for OPXV testing. He was started on oral tecovirimat empirically, after which all test results from swabs of skin and eye lesions returned PCR-positive for OPXV. The patient also received topical trifluridine for 5 days and antibacterial drops to the right eye, as well as intravenous antibiotics for preseptal cellulitis. He was discharged upon clinical improvement 5 days after admission, to complete a 14-day course of oral tecovirimat.

## Patient E

In July 2022, a previously healthy woman aged 30–39 years was evaluated for pustular lesions on her vaginal labia (Table) (Figure 1). A swab of those lesions tested PCR-positive for OPXV. During the week after symptom onset, lesions spread to her back, buttocks, chin, forehead, and left lower eyelid. She began experiencing left eye pain and redness. She sought medical care after noticing a lesion on the globe of her left eye, for which she was admitted to a hospital. Ophthalmologic exam was notable for left eye conjunctivitis, a bulbar conjunctival lesion, and a subconjunctival nodule. She did not experience vision changes. Tecovirimat was not immediately available, and she was treated with topical trifluridine to the left eye. Her ocular symptoms improved, and she was discharged after 3 days with a 14-day course of oral tecovirimat and 5 more days of topical trifluridine.

## Discussion

This report highlights the varying clinical manifestations of ocular monkeypox and the importance of prompt evaluation and treatment to prevent sight-threatening complications. All five patients with ocular monkeypox described in this report suffered prolonged illness, four were hospitalized, and one experienced significant vision impairment. Two patients had HIV-associated immunocompromise and experienced delays in initiation of treatment for monkeypox. One of these patients experienced vision loss; he remains in treatment and his prognosis for vision recovery is currently unknown. Urgent referral for ophthalmologic evaluation and prompt antiviral therapy should be considered for patients with monkeypox and ocular signs or symptoms (e.g., vision changes or eye pain, itching, redness, swelling, or foreign body sensation) or lesions near the eye. Clinicians should consider initiation of prompt systemic antiviral therapy as well as topical trifluridine for patients with ocular monkeypox.^¶¶^

Several strategies might help prevent ocular monkeypox and associated complications. To decrease the risk for autoinoculation, persons with monkeypox should be advised to practice hand hygiene and to avoid touching their eyes, which includes refraining from using contact lenses (*2*). Short turnaround times for OPXV/MPXV PCR test results might help prevent delays in treatment initiation. For persons with suspected ocular monkeypox, or for persons with suspected monkeypox who are at risk for severe manifestations of the disease (e.g., those with HIV-associated immunocompromise), clinicians might consider initiating empiric treatment for monkeypox while test results are pending. Health care providers can contact their public health jurisdictions and CDC for support on the use of medical countermeasures to treat patients with monkeypox*** (*3*).

The findings in this report are subject to at least two limitations. First, the cases described might not be representative of patients with ocular monkeypox in the United States, and conclusions cannot be drawn about the frequency of reported events. Although the frequency of ocular monkeypox during the current outbreak is unknown, national surveillance data from the United States suggest that 5% of patients with monkeypox report ocular symptoms^†††^ (*4*). Second, not every patient underwent testing of ocular lesions for OPXV/MPXV or exhaustive testing for other ocular infections. However, the clinical findings in these patients were compatible with descriptions of ocular monkeypox from other studies (*5*,*6*).

Ocular monkeypox is a potentially sight-threatening infection. Urgent ophthalmologic evaluation and the provision of timely medical countermeasures for patients with suspected or confirmed ocular monkeypox might help prevent poor outcomes.

**Summary**

**What is already known about this topic?**

Patients with monkeypox can experience serious ocular complications, which are not well described during the current outbreak.

**What is added by this report?**

This report describes five cases of ocular monkeypox identified in the United States during July–September 2022. Patients with ocular monkeypox, including those with HIV-associated immunocompromise, have experienced delays in treatment initiation, prolonged illness, hospitalization, and vision impairment.

**What are the implications for public health practice?**

Health care providers and public health practitioners should be aware that ocular monkeypox, although rare, is a sight-threatening condition. Patients with signs and symptoms compatible with ocular monkeypox should be considered for urgent ophthalmologic evaluation and treatment. Prompt notification of public health officials can help support these efforts.

## Footnotes

* Case counts included confirmed and probable monkeypox cases. https://www.cdc.gov/poxvirus/monkeypox/response/2022/us-map.html (Accessed October 11, 2022).

† Tecovirimat, an FDA-approved treatment for smallpox, is an antiviral drug that has demonstrated effectiveness in animal MPXV models. Interim CDC guidance currently recommends that tecovirimat be considered in patients with severe monkeypox, those at high risk for severe disease, or those whose infection involves accidental implantation in the eyes or other sensitive anatomic areas where monkeypox might constitute a special hazard. https://www.cdc.gov/poxvirus/monkeypox/clinicians/Tecovirimat.html

§ Trifluridine, an antiviral drug used to treat herpes simplex keratitis, can be considered to treat conjunctivitis and keratitis caused by MPXV, in consultation with an ophthalmologist. Trifluridine is a preferred treatment for ocular infection with vaccinia virus, which can occur as a complication of autoinoculation after vaccination with ACAM 2000. https://www.cdc.gov/poxvirus/monkeypox/clinicians/ocular-infection.html

¶ CDC is offering a monkeypox clinical consultation service during the ongoing monkeypox outbreak. Health care providers seeking additional clinical guidance can contact CDC Emergency Operations by phone (770-488-7100) or by email (eocevent482@cdc.gov).

** Case definitions for use in the 2022 monkeypox response. https://www.cdc.gov/poxvirus/monkeypox/clinicians/case-definition.html

†† 45 C.F.R. part 46.102(l)(2), 21 C.F.R. part 56; 42 U.S.C. Sect. 241(d); 5 U.S.C. Sect. 552a; 44 U.S.C. Sect. 3501 et seq.

§§ Clinical recognition, monkeypox. https://www.cdc.gov/poxvirus/monkeypox/clinicians/clinical-recognition.html

¶¶ Interim clinical considerations for management of ocular monkeypox virus infection. https://www.cdc.gov/poxvirus/monkeypox/clinicians/ocular-infection.html

*** Interim clinical guidance for the treatment of monkeypox. https://www.cdc.gov/poxvirus/monkeypox/clinicians/treatment.html#anchor_1655488233196

††† This frequency is lower than that reported from the Democratic Republic of the Congo, where 23% of patients with monkeypox had conjunctivitis, a difference that might be related to the virulence of the MPXV clade causing infection during the ongoing outbreak, or to epidemiologic or clinical factors. https://www.cdc.gov/poxvirus/monkeypox/clinicians/ocular-infection.html
