# *Erratum:* Vol. 71, No. 42

**DOI:** 10.15585/mmwr.mm7148a5

**Published:** 2022-12-02

In the report, “Ocular Monkeypox — United States, July–September 2022,” the case report of patient E should have included a citation to a previously published case report that described the first 2 days of patient E's clinical course. On p. 1346, the last two sentences under the heading “Patient E” should have read “**Neither tecovirimat nor trifluridine was immediately available; the patient was treated with naproxen**. Her ocular symptoms improved, and she was discharged after 3 days with a 14-day course of oral tecovirimat and **a 5-day course of topical trifluridine (*2*)**. In the figure on p. 1344 (Figure 1), the timeline of treatment administration for patient E should have indicated 5 days of treatment with trifluridine. In addition, on p. 1347, the list of references should have included the following: “**2. Foos W, Wroblewski K, Ittoop S. Subconjunctival nodule in a patient with acute monkeypox. JAMA Ophthalmol 2022;140:e223742**.”

**FIGURE 1:**
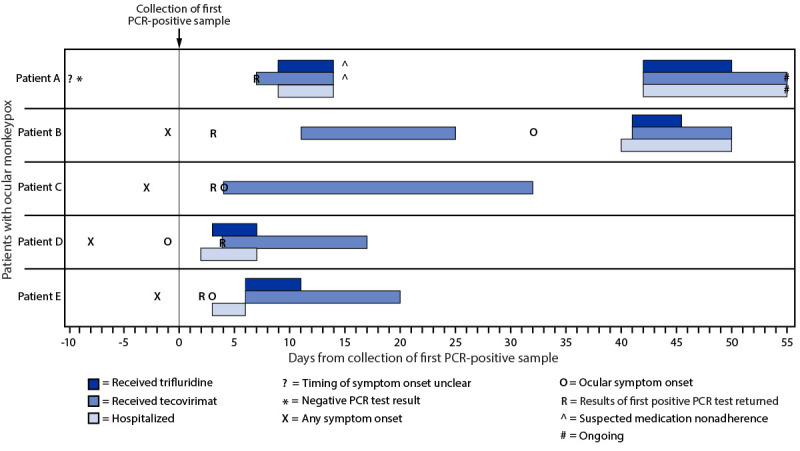
Timeline of testing, symptom onset, and initiation of medical countermeasures for patients with ocular monkeypox — United States, July–September 2022 **Abbreviation:** PCR = polymerase chain reaction.

